# Urokinase-type plasminogen activator receptor (uPAR) as a therapeutic target in cancer

**DOI:** 10.1186/s12967-022-03329-3

**Published:** 2022-03-18

**Authors:** Bing-Tao Zhai, Huan Tian, Jing Sun, Jun-Bo Zou, Xiao-Fei Zhang, Jiang-Xue Cheng, Ya-Jun Shi, Yu Fan, Dong-Yan Guo

**Affiliations:** 1 State Key Laboratory of Research & Development of Characteristic Qin Medicine Resources (Cultivation), and Shaanxi Key Laboratory of Chinese Medicine Fundamentals and New Drugs Research, and Shaanxi Collaborative Innovation Center of Chinese Medicinal Resources Industrialization, Shaanxi University of Chinese Medicine, Xi’an, 712046 China; 2 Xi’an Hospital of Traditional Chinese Medicine, Xi’an, 710021 China

**Keywords:** Urokinase-type plasminogen activator receptor (uPAR), Nanoparticles (NPs), Photodynamic therapy (PDT)/photothermal therapy (PTT), Oncolytic virotherapy, Gene therapy technologies, Monoclonal antibody therapy, Tumour immunotherapy

## Abstract

Urokinase-type plasminogen activator receptor (uPAR) is an attractive target for the treatment of cancer, because it is expressed at low levels in healthy tissues but at high levels in malignant tumours. uPAR is closely related to the invasion and metastasis of malignant tumours, plays important roles in the degradation of extracellular matrix (ECM), tumour angiogenesis, cell proliferation and apoptosis, and is associated with the multidrug resistance (MDR) of tumour cells, which has important guiding significance for the judgement of tumor malignancy and prognosis. Several uPAR-targeted antitumour therapeutic agents have been developed to suppress tumour growth, metastatic processes and drug resistance. Here, we review the recent advances in the development of uPAR-targeted antitumor therapeutic strategies, including nanoplatforms carrying therapeutic agents, photodynamic therapy (PDT)/photothermal therapy (PTT) platforms, oncolytic virotherapy, gene therapy technologies, monoclonal antibody therapy and tumour immunotherapy, to promote the translation of these therapeutic agents to clinical applications.

## Background

Urokinase-type plasminogen activator receptor (uPAR), also known as CD87, is encoded by the PLAUR gene and belongs to the lymphatic antigen-6 superfamily [1, 2]. uPAR was first identified as the cell surface receptor for urokinase plasminogen activator (uPA) in 1985 [3, 4]. The mature uPAR molecule is a single-chain membrane glycoprotein receptor composed of 313 amino acid residues and is anchored to the cell membrane by a glycosylphosphatidylinositol (GPI) linkage; it contains 3 homologous domains, D1, D2 and D3, with a total molecular weight of 55–60 kDa [5, 6]. uPAR mediates a variety of biological processes, such as plasminogen activation, proteolysis, cellular signal transduction and adhesion [7–9]. Under normal physiological conditions, uPAR is usually expressed at a low level. In the processes of tissue remodelling, wound healing, inflammation and embryogenesis, uPAR is transiently expressed at high levels and participates in the processes of extracellular matrix (ECM) degradation, thrombolysis, cell invasion and migration [10–14].

Classically, the function of uPAR is to act as a receptor for the zymogen form of uPA (pro-uPA) and trigger a cascade of proteolytic events that leads to the degradation of ECM [15, 16]. Once pro-uPA is activated to uPA, it converts plasminogen to its active form, plasmin, which activates downstream proteases such as pro-matrix metalloproteinase (MMP)-3 and MMP-3, pro-MMP-9 and MMP-9, leading to ECM remodelling [17–19]. Plasmin is also able to release ECM bound growth factors that contribute to tumour progression [20, 21].

In addition to its proteolytic role, uPAR interacts with vitronectin (Vn) [22] and transmembrane receptors, including integrins (α5β1, α3β1, αvβ3 and αvβ5) [23–27] and receptor tyrosine kinases [the epidermal growth factor receptor (EGFR) and platelet-derived growth factor receptor (PDGFR), G-protein coupled receptors (GPCRs), very low-density lipoprotein receptor (VLDLR) family members], thereby activating intracellular focal adhesion kinase (FAK) signalling, regulating intracellular pathways [Ras/mitogen-activated protein kinase (MAPK), Ras-related C3 botulinum toxin substrate 1 (Rac1)/MAPK, phosphatidylinositol 3-kinase (PI3K)/protein kinase B (AKT), and Janus-associated kinase 1 (JAK1)], and triggering cellular responses such as cell migration, adhesion, proliferation, angiogenesis and the epithelial–mesenchymal transition (EMT) [28–36]. Moreover, the cleaved form of uPAR (D2–D3 fragment), interacts with members of the formyl peptide receptor (FPR) family of GPCRs via its exposed N-terminal _88_SRSRY_92_ sequence, initiating both angiogenic and inflammatory processes [37, 38].

**Fig. 1:**
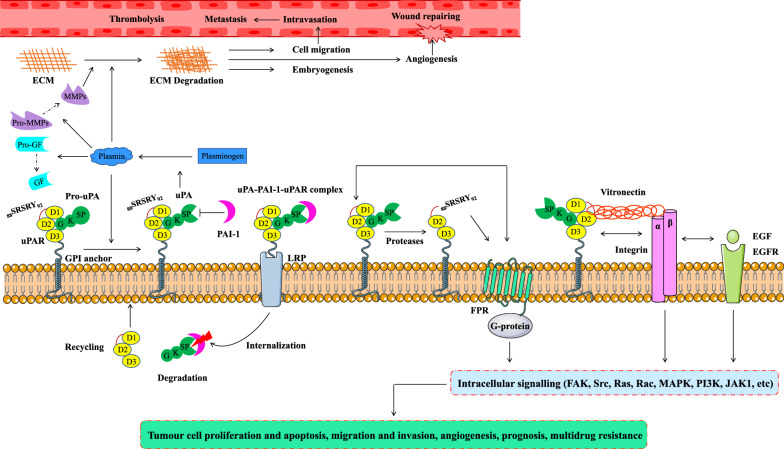
Schematic representation of the uPAR-mediated pathways. The GPI-anchored receptor uPAR consisting of D1, D2, and D3 domains binds the zymogen pro-uPA and the active uPA through the GF domain. The active form of uPA then converts plasminogen into plasmin, which subsequently cleaves and activates GFs, and MMPs, leading to the degradation of ECM in important physiological processes and in pathological processes associated with cancer development. PAI-1 inhibits the catalytic activity of both uPA and plasmin. Internalization and recycling of uPAR occur after a uPA-PAI-1-uPAR complex has formed, resulting in the degradation of uPA-PAI-1 and the recycling of uPAR to the cell surface. uPAR is cleaved between the D1 and D2 domains, exposing the _88_SRSRY_92_ sequence at its N-terminus to interact with FPR of the GPCRs, promoting its internalization and activating signalling. In addition to uPA, uPAR interacts with Vn, integrins and other cell surface receptors, such as EGFRs, to activate different intracellular signalling pathways [FAK, Src, Ras, Rac, MAPK, PI3K, JAK1, etc.] and regulate tumour cell proliferation and apoptosis, migration and invasion, angiogenesis, prognosis and multidrug resistance. *uPAR* urokinase-type plasminogen activator receptor, *uPA* urokinase plasminogen activator, *GPI* glycosylphosphatidylinositol, *GF* growth factor, *MMPs* matrix metalloproteinases, *ECM* extracellular matrix, *PAI-1* plasminogen activator inhibitor-1, *Vn* vitronectin, *EGFR* epidermal growth factor receptor, *LRP* low-density lipoprotein receptor-related protein, *GPCRs* G-protein coupled receptors, *FPR* formyl peptide receptor, *FAK* focal adhesion kinase, *Src* tyrosine-protein kinase, *MAPK* mitogen activated protein kinase, *Rac* Ras-related C3 botulinum toxin substrate, *PI3K* phosphatidylinositol 3-kinase, *JAK1* janus kinase 1

Finally, uPAR is also involved in the internalization of the uPA-plasminogen activator inhibitor (PAI)-1-uPAR complex, degradation of uPA-PAI-1, and recycling of unoccupied uPAR. When uPA-uPAR is inactivated by PAI-1, internalization via low-density lipoprotein receptor related protein (LRP) is initiated, leading to clathrin-mediated endocytosis of the uPA-PAI-1-uPAR complex. Once internalized, uPA-PAI-1 dissociates from uPAR and is trafficked to the lysosome for degradation, while the unoccupied uPAR is recycled to the cell surface [39–41]. A schematic representation of the uPAR-mediated pathways is shown in Fig. 1.

In recent years, many studies have shown that uPAR is closely related to the invasion and metastasis of malignant tumours. uPAR plays important roles in the degradation of ECM, tumour angiogenesis, cell proliferation and apoptosis, is related to the multidrug resistance (MDR) of tumour cells, and has important guiding significance for the judgement of tumour malignancy and prognosis. In this review, we summarize the new application of uPAR as a target of nanoplatforms carrying therapeutic agents, photodynamic therapy (PDT)/photothermal therapy (PTT) platforms, oncolytic virotherapy, gene therapy technologies, monoclonal antibody therapy and tumour immunotherapy to promote the translation of these therapeutic agents to clinical applications.

## uPAR in cancer progression

uPAR has multiple functional roles associated with tumour progression, including tumour proliferation and apoptosis, metastasis, angiogenesis, MDR and prognosis. An analysis of tumour samples has shown high uPAR expression in most solid tumour tissues, such as breast [42], lung [43], bladder [44], ovarian [45], prostate [46], liver [47], colon [48], pancreatic [49] and gastric cancer [50] as well as gliomas [51] and several haematologic malignancies [52, 53]. Moreover, uPAR is expressed at high levels on stromal cells in the tumour microenvironment, such as vascular endothelial cells, tumour-related fibroblasts and tumour-related macrophages, and its expression level is closely related to tumour aggressiveness and the survival of patients with tumours [54–57]. Therefore, treatments targeting uPAR expressed on tumour-associated stromal cells may be as important as treatments targeting uPAR expressed on tumour cells and may lead to enhanced antitumour activity.

uPAR interacts with a variety of surface transmembrane proteins, such as integrins and EGFR, thereby activating intracellular FAK, extracellular regulatory protein kinase (ERK) and MAPK signalling to inhibit cell apoptosis and promote cell proliferation. For example, the interaction between uPAR and a5β1 integrin activates EGFR through a FAK-dependent pathway, which subsequently activates the ERK signalling pathway and promotes cell proliferation [58]. Inhibition of uPAR expression destroy the uPAR/integrin interaction and inhibits the MAPK pathway to arrest Hep3 cells in G0/G1 phase [59]. The suppression of uPAR expression in vitro by transfection inhibits the proliferation of meningioma cells by downregulating transforming growth factor-β (TGF-β) 1 expression [60], arrests glioma SNB19 cells in G2 phase and increases caspase-dependent cell apoptosis [61]. Moreover, inhibiting the expression of uPAR in vitro by transfection promotes the apoptosis of human melanoma cells by increasing the expression of the p53 protein and activating the apoptosis pathway mediated by retinoic acid inducible gene 1 (RIG-1) [62].

Inhibition of uPAR expression prevents tumour invasion and migration. For example, inhibiting the expression of uPA/uPAR blocks the invasion of glioma SNB19 cells by reducing Ras mediated phosphorylation of FAK, p38MAPK, c-Jun N-terminal kinase (JNK) and ERK1/2 and MAPK kinase (MEK) activation of the PI3K/AKT/mammalian target of rapamycin (mTOR) signalling pathway [63]. Inhibition of uPA/uPAR expression also prevents the invasion of glioma cells by inhibiting Notch-1 receptor cleavage, signal transduction and endosomal transport [64]. Treatments targeting uPAR in human pancreatic cancer cells inhibit the migration and invasion of mouse tumour cells mediated by c-met and insulin like growth factor 1 receptor (IGF1R) [65]. Inhibition of uPAR expression along with the expression of uPA, human epidermal growth factor receptor-2 (HER-2), or IGF1R or in combination with trastuzumab further inhibits the invasion and migration of different breast cancer cell lines [66–68].

Angiogenesis is the process of forming new blood vessels from existing blood vessels. It plays a vital role in tumour growth, invasion and metastasis. uPAR also promotes tumour angiogenesis. For example, uPAR promotes angiogenesis by inhibiting the expression of phosphatase and tensin homologue deleted on chromosome 10 (PTEN) [69]. In endothelial cells and glioblastoma cells, silencing the expression of uPA/uPAR inhibits tumour angiogenesis by increasing the expression of tissue inhibitor of matrix metalloproteinase-1 (TIMP-1) and increasing the secretion of soluble vascular endothelial growth factor (VEGF) receptor (VEGFR) 1 (SVEGFR1) [70]. Herkenne et al. also found that knockout of uPAR in human umbilical vein endothelial cells (HUVECs) blocks VEGFR2 signalling, thereby preventing VEGF-induced angiogenesis [71].

High levels of uPAR expression have been detected in a variety of cancer cells but very low levels are present in normal cells, indicating that the level of uPAR in tumour tissue is closely related to the tumour malignancy and prognosis of patients with cancer [72]. Elevated levels of uPAR are observed in prostate cancer, correlating with increased aggressiveness, postoperative progression and metastasis [73, 74]. In another study, Memarzadeh et al. found that the expression of uPAR in surgically removed endometrial tissue was positively correlated with the malignancy of endometrial cancer [75]. A study using 45 fresh tumour tissues observed the presence of uPAR in 1/3 of melanomas [76]. Yang et al. suggested that uPAR is useful as an independent prognostic factor for the survival and metastasis of patients with colorectal cancer [77]; Halamkova et al. also reported a correlation between uPAR expression and the grade of colorectal cancer [78]. Many studies have shown increased levels of uPAR and their related to liver metastasis and a poor prognosis for patients with hepatocellular carcinoma (HCC) [79–81]. According to Chen et al., the levels of uPAR in patients with lung cancer are significantly increased [82]. A study has shown an association between an increased level of the uPAR D1 domain and shorter overall survival of patient with small cell lung cancer [83]. uPAR expression in tumour tissues is also significantly increased in non-small cell lung cancer (NSCLC) [84]. In gastric cancer, increased uPAR expression and decreased uPAR expression are related to a poor prognosis and prolonged patient survival, respectively [85, 86]. In oral squamous cell carcinoma (OSCC), the levels of uPAR are elevated, and a strong correlation between the expression of uPAR and the aggressiveness of the tumour has been identified [87]. Increased uPAR levels are closely related to a poor prognosis for patients with bladder cancer [88, 89]. High levels of uPAR are present in 94% of muscle-invasive bladder cancer and 54–71% of nonmuscle-invasive bladder cancer, but the protein is almost undetectable in healthy bladder tissue [90]. The expression of uPAR is significantly increased in laryngeal squamous cell carcinoma, which may help increase invasion and metastasis [91]. In acute myeloid leukaemia (AML), the high expression of uPAR is also associated with the aggressiveness of the disease [92]. Therefore, the expression level of uPAR may be an important marker for judging the degree of malignancy and the survival of patients.

An association between uPAR expression and the MDR of tumour cells has also been identified. Drug resistance is an important cause of the failure of tumour treatment. A study has shown that inhibition of uPAR in vitro promotes the apoptosis of melanoma cells resistant to B-RAF inhibitors and MEK inhibitors by increasing the level of Noxa [62]. High uPAR expression may allow head and neck squamous cell carcinoma, small cell lung cancer, and malignant pleural mesothelioma to develop resistance to chemotherapy [93–95]. uPAR enhances the resistance of breast cancer to tamoxifen by activating ERK1/2 [96], and renders NSCLC resistant to gefitinib by activating the EGFR/pAKT/survivin signalling pathway [97]. Inhibition of uPAR expression reduces the resistance of mouse brain neuroma cells to 5-fluorouracil (5-FU), cisplatin (Cis), docetaxel (DTX) and doxorubicin (Dox) [98]. Laurenzana et al. showed that BRAF-mutated melanoma cells with different uPAR expression levels have different sensitivities to verofenil; high levels of uPAR decrease the sensitivity of BRAF-mutated melanoma cells to verofenil, while a reduction in uPAR expression restores the sensitivity of drug-resistant cells to verofenil [99]. As shown in the study by LeBeau et al., MCF-7 cells resistant to tamoxifen and MDA-MB-231 cells resistant to Dox and paclitaxel (PTX) exhibit markedly higher expression of uPAR than parental MCF-7 and MDA-MB-231 cells, respectively [100].

In summary, the dysregulation of uPAR plays a key role in tumour progression. Given the broad expression of uPAR by a variety of different tumour types and the selective expression of uPAR by tumour cells and tumour-related stromal cells in the tumour microenvironment compared to normal cells, uPAR is an attractive target for the treatment of tumours.

## Targeting uPAR for antitumour therapy

Compared with normal tissues, high uPAR expression in tumours has been shown, and thus researchers have proposed uPAR as a therapeutic target and a targeting agent for the treatment of cancer [101]. Over the past 30 years, a variety of therapeutic agents that target uPAR have been developed to treat cancer. For example, peptides AE105 (D-Cha-F-s-r-Y-L-W-S) [102], AE120 ([D-Cha-F-s-r-Y-L-W-S]2-βA-K) [102], Å6 (Ac-KPSSPPEE-Am) [103], ATF [104], and U11 (VSNKYFSNIHW) [105], and the cyclic peptides cyclo^19,31^uPA_19–31_ [106], cyclo^19,31^[D-Cys^19^]-uPA_19–31_ [107], WX-360 (cyclo^21,29^[D-Cys21]-uPA_21–30_[S21C;H29C]) and WX-360-Nle (cyclo^21,29^[D-Cys21]-uPA_21–30_[S21C;K23Nle;H29C]) [108] block the uPA/uPAR interaction. Peptides M25 (PRYQHIGLVAMFRQNTG) [109], α325 (PRHRHMGAVFLLSQEAG) [110], p25 (AESTYHHLSLGYMYTLN-NH_2_) [111], m.P243-251 (TASWCQGSH) [112], D2A-Ala (IQEGAAGRPKDDR) [113] and polyethylene glycol (PEG)ylated D2A-Ala peptide (PEG-D2A-Ala) [114] inhibit the uPAR/integrin or uPAR/Vn interaction. Peptides pyro glutamic acid (pGlu)-Arg-Glu-Arg-Tyr-NH_2_ (pERERY-NH_2_) [115], RERF (Ac-Arg-Glu-Arg-Phe-NH_2_) [116], UPARANT (Ac-L-Arg-Aib-L-Arg-D-Ca(Me)Phe-NH_2_) [117], cyclic SRSRY peptide ([SRSRY]) [118], and RI-3 [Ac-(D)-Tyr-(D)-Arg-Aib-(D)-Arg-NH_2_] [119] block the interaction of SRSRY and *N*-formyl-Met-Leu-Phe (fMLF) with the FPR family of GPCRs. Human and mouse uPA1-48 (huPA1-48 and muPA1-48), human and murine uPA1-48 fusion proteins (huPA1-48Ig and muPA1-48Ig) [120], and human and mouse pegylated uPA1-48 (PEGh1-48 and PEGhm1-48) [121] also inhibit tumour growth by inhibiting tumour stromal cell uPAR-dependent plasminogen activation. The small-molecule inhibitors IPR-456 [122], IPR-803 [123], IPR-3011 [124], IPR-3577 [125], 7 [126], LLL-1fsi [127], MS#479 [2-(pyridin-2-ylamino)-quinolin-8-ol] and MS#305 [2,2′-(methylimino)di (8-quinolinol)] [128], Compounds 6 and 37 [129], and docosahexaenoic acid (DHA) [130] inhibit the uPAR/uPA, uPAR/integrin, uPAR/Vn or uPAR/FPR interaction. The ligand-targeted toxins DTAT [diphtheria toxin (DT) and ATF] [131, 132], DTATEGF (ATF, EGF and DT) [133], DTAT13 [ATF, interleukin-13 (IL-13) and DT] [134, 135], eBAT (EGFATFKDEL 7mut) [136–141], ATF-SAP (ATF and Saporin) [142, 143], PAI-2-*N*-AIE conjugate [5,7-dibromo-*N*-(*p*-hydroxymethylbenzyl)isatin and PAI-2] [144], DTU2GMCSF [DT and granulocyte–macrophage colony-stimulating factor (GM-CSF)] [145], ATF-PE38 and ATF-PE38KDEL [ATF and *Pseudomonas exotoxin* A (PE38)] [146] exert antitumor effects by targeting uPAR and releasing toxins. The uPAR-targeted peptides, small-molecule inhibitors and ligand-targeted toxins are summarized in Table 1.

**Table 1:** The uPAR-targeted peptides, small-molecule inhibitors and ligand-targeted toxins

Peptides/small-molecule inhibitors/ligand-targeted toxins	Sequence/structure/composition	Action site/target	References
AE105	D-Cha-F-s-r-Y-L-W-S	uPA/uPAR	[102]
AE120	[D-Cha-F-s-r-Y-L-W-S]2-βA-K	uPA/uPAR	[102]
Å6	Ac-KPSSPPEE-Am	uPA/uPAR	[103]
ATF	An amino-terminal fragment of urokinase with EGF-like domain and kringle domain	uPA/uPAR	[104]
U11	VSNKYFSNIHW	uPA/uPAR	[105]
A stable disulfide-bridged cyclic form of the linear peptide uPA_19–31_	cyclo^19,31^uPA_19–31_	uPA/uPAR	[106]
A peptide variant of cyclo^19,31^uPA_19–31_	cyclo^19,31^[D-Cys^19^]-uPA_19–31_	uPA/uPAR	[107]
WX-360	cyclo^21,29^[D-Cys21]-uPA_21–30_[S21C;H29C]	uPA/uPAR	[108]
WX-360-Nle	cyclo^21,29^[D-Cys21]-uPA_21–30_[S21C;K23Nle;H29C]	uPA/uPAR	[108]
M25	PRYQHIGLVAMFRQNTG	uPAR/β1-integrins	[109]
α325	PRHRHMGAVFLLSQEAG	uPAR/Vn	[110]
p25	AESTYHHLSLGYMYTLN-NH_2_	uPAR-integrin uPAR/Vn	[111]
m.P243-251	TASWCQGSH	uPAR/integrin α5β1	[112]
D2A-Ala	IQEGAAGRPKDDR	uPAR/integrin avβ3/a5β1	[113]
PEGylated D2A-Ala	PEG-D2A-Ala	uPAR/integrin avβ3/a5β1	[114]
pERERY-NH_2_	Pyro glutamic acid (pGlu)-Arg-Glu-Arg-Tyr-NH_2_	fMLF/FPR	[115]
RERF	Ac-Arg-Glu-Arg-Phe-NH_2_	SRSRY/FPR fMLF/FPR	[116]
UPARANT	Ac-L-Arg-Aib-L-Arg-D-Ca(Me)Phe-NH_2_	fMLF/FPR	[117]
cyclic SRSRY peptide ([SRSRY])	[Ser-Arg-Ser-Arg-Tyr]^§^	SRSRY/FPR1 fMLF/FPR1	[118]
RI-3	Ac-(D)-Tyr-(D)-Arg-Aib-(D)-Arg-NH_2_	fMLF/FPR1	[119]
huPA1-48 and muPA1-48	The growth factor domains of human and murine urokinase	Tumour stromal cell uPAR dependent plasminogen activation	[120]
huPA1-48Ig and muPA1-48Ig	Modify huPA1-48 and muPA1-48 with the constant region of human IgG_1_	Tumour stromal cell uPAR dependent plasminogen activation	[120]
PEGh1-48 and PEGhm1-48	Human and mouse pegylated uPA1-48	Tumour stromal cell uPAR dependent plasminogen activation	[121]
IPR-456		uPA/uPAR	[122]
IPR-803		uPA/uPAR	[123]
IPR-3011		uPA/uPAR	[124]
IPR-3577		uPA/uPAR	[125]
7		uPAR/uPA_ATF_ uPAR/Vn	[126]
LLL-1fsi		uPA/uPAR	[127]
MS#479 [2-(Pyridin-2-ylamino)-quinolin-8-ol]		uPAR/integrin	[128]
MS#305 [2,2′-(methylimino)di (8-quinolinol)]	〹	uPAR/integrin	[128]
Compounds 6		uPAR/Vn uPAR/FPR	[129]
Compounds 37		uPAR/Vn uPAR/FPR	[129]
Docosahexaenoic acid (DHA)		suppress uPAR expression	[130]
DTAT	DT and ATF	uPAR	[131, 132]
DTATEGF	ATF, EGF and DT	uPAR, EGFR	[133]
DTAT13	ATF, IL-13 and DT	uPAR, IL-13 receptors	[134, 135]
eBAT (EGFATFKDEL 7mut)	ATF, EGF, truncated PE38 with a terminal lysyl-aspartyl-glutamyl-leucine (KDEL) sequence and eight amino acids representing the seven major epitopes on PE38 were mutated	uPAR, EGFR	[136–141]
ATF-SAP	ATF and SAP	uPAR	[142, 143]
PAI-2-N-AIE	PAI-2 and *N*-AIE	uPAR	[144]
DTU2GMCSF	DT, GM-CSF and uPA	uPAR, GM-CSF receptor	[145]
ATF-PE38	ATF and PE38	uPAR	[146]
ATF-PE38KDEL	ATF and PE38 with a terminal KDEL sequence	uPAR	[146]

uPA: urokinase plasminogen activator; uPAR: urokinase-type plasminogen activator receptor; Vn: vitronectin; PEG: polyethylene glycol; fMLF: *N*-formyl-Met-Leu-Phe; FPR: formyl peptide receptor; DT: diphtheria toxin; IL-13: interleukin-13; PE38: *Pseudomonas exotoxin* A; EGF: epidermal growth factor; EGFR: epidermal growth factor receptor; SAP: Saporin; PAI-2: plasminogen activator inhibitor type 2; *N*-AIE: 5,7-dibromo-*N*-(*p*-hydroxymethylbenzyl)isatin was conjugated to PAI-2 via an esterase-labile succinate linker; GM-CSF: granulocyte-macrophage colony-stimulating factor

However, although research has been conducted for more than 30 years, none of these treatments have advanced into clinical application. The pleiotropic nature of uPAR interactions and function, uPAR structural flexibility, species specificity of the uPA-uPAR interaction, limitations of tumour models, the characteristic that uPAR expression is increased on tumour cells and tumour-associated stromal cells, and the baseline expression of uPAR in the glomeruli of normal kidneys that may result in potential “on-target off-tumour” toxicity are all the main hurdles to the development of uPAR inhibitors [72, 101, 147–152]. Furthermore, linear peptides based on the sequence of uPA lack potency and have poor pharmacological properties and stability due to susceptibility to exoprotease degradation in the plasma [153]; screening for small-molecule inhibitors is inefficient due to a lack of detailed structural information on the interactions of uPAR with its binding partners such as integrins [154–156]. Some uPAR-targeted small-molecule inhibitors are hydrophobic and have limited bioavailability [123, 125, 157]; and due to the large surface area at the protein–protein interface, the development of small molecules specifically targeting this flexible hydrophobic cavity in uPAR also represent a challenging task [129, 158]. Similarly, ligand-targeted toxins must overcome many barriers before they reach human clinical trials, including determining the appropriate dosing strategy and sequence of administration, increasing the potency and reducing the immunogenicity of the toxin [159, 160].

**Fig. 2:**
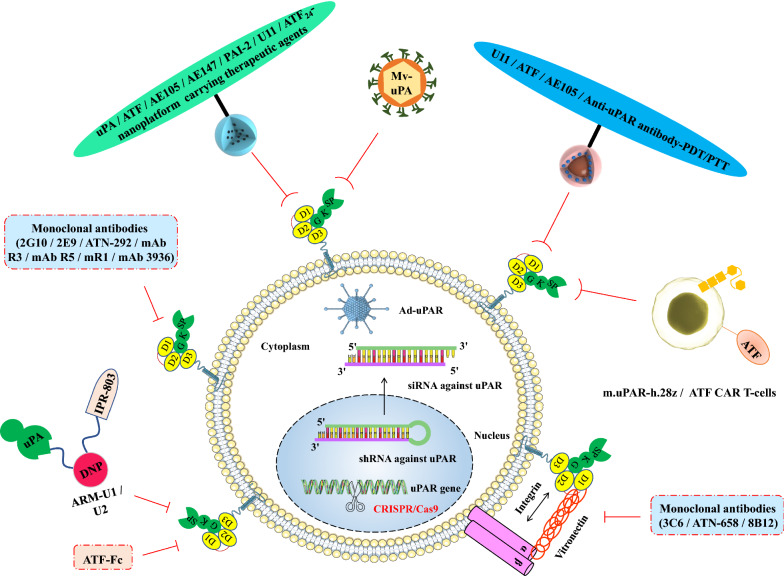
uPAR was used as a target in nanoplatforms carrying therapeutic agents, PDT/PTT platforms, oncolytic virotherapy, gene therapy techniques, monoclonal antibody therapy and tumour immunotherapy to enhance antitumor effects. (1) uPAR-targeted nanoplatforms carrying therapeutic agents have great potential for the development of targeted therapeutic and imaging approaches that are capable of enhancing the therapeutic effect of nanoparticle drugs on various cancers. (2) uPAR-targeted PDT/PTT platforms may be regarded as promising cancer therapeutic strategies due to their unique advantages such as minor trauma, improved selectivity and reduced side effects. (3) uPAR-targeting oncolytic measles virus (MV-uPA) is an innovative biological strategy associated with potent antitumour effects. (4) uPAR-targeted gene therapy techniques using adenovirus-mediated antisense uPAR therapy, RNA interference (RNAi) technology and novel CRISPR/Cas9 gene editing technology may represent useful tools and provide new therapeutic options for aggressive cancers. (5) uPAR-targeted monoclonal antibody therapy may provide new breakthroughs in the development of anticancer therapy. (6) uPAR-targeted CAR T-cell immunotherapy and ARMs have the ability to target uPAR-expressing cancers for immune-mediated cell death. *PDT/PTT* photodynamic therapy/photothermal therapy, *MV-uPA* uPAR-targeting oncolytic measles virus, *RNAi* RNA interference, *CRISPR/Cas9* RNA-guided clustered regularly interspaced short palindromic (CRISPR) in combination with a CRISPR-associated nuclease 9 (Cas9) nuclease system, *CAR* chimeric antigen receptor, *ARMs* antibody-recruiting molecules

In recent years, with the interdisciplinary integration of cell biology and materials science, many innovative tumour-targeted therapeutic technologies targeting uPAR have emerged, providing new development directions for precise and efficient tumour therapy. uPAR-targeted nanoplatforms carrying therapeutic agents have great potential in enhancing active tumour targeting, improving delivery efficiency, reducing drug toxicity, increasing the hydrophilicity of hydrophobic drugs, achieving tumour diagnosis and treatment integration, and in multimodal synergistic antitumor applications. uPAR-targeted PDT/PTT platforms may be regarded as promising cancer therapeutic strategies due to their unique advantages such as minor trauma, improved selectivity and reduced side effects. uPAR-targeting oncolytic measles virus (MV-uPA) is an innovative biological strategy associated with potent antitumour effects. uPAR-targeted clustered regularly interspaced short palindromic (CRISPR)/CRISPR-associated protein-9 nuclease (Cas9) gene-editing technology may provide new therapeutic trearments for aggressive cancers. uPAR-targeted monoclonal antibody therapy may provide new breakthroughs for the development of anticancer therapy. uPAR-targeted chimeric antigen receptor (CAR) T-cell immunotherapy and antibody-recruiting molecules (ARMs) have the ability to target uPAR-expressing cancers for immune-mediated cell death. Therefore, this review focuses on some new applications of uPAR in the six fields described above (Fig. 2).

## uPAR-targeted nanoplatforms carrying therapeutic agents

**Fig. 3:**
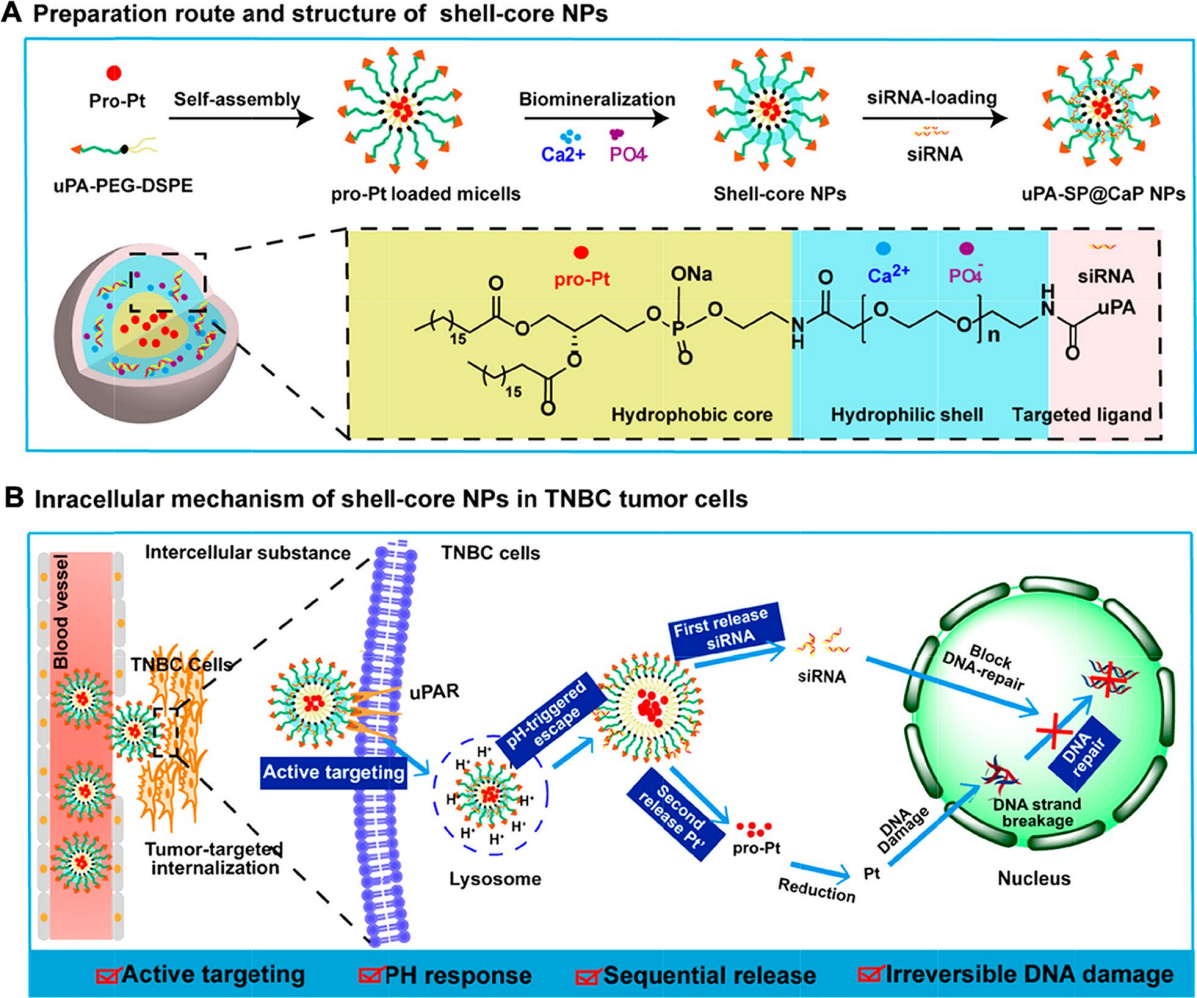
Integration of siRNA and Pro-Pt into uPA peptide-targeted multifunctional shell-core NPs for the synergistic treatment of TNBC. **A** The tumour-targeted CaP shell-core NPs were prepared using the biomineralization method, where the organic DSPE-PEG-uPA core encapsulates the chemotherapeutic agent Pro-Pt (Pt′) followed by negatively charged siRNA adsorbing in the inorganic porous CaP shell. **B** The intracellular mechanism of uPA-SP@CaP NPs in TNBC cells. (i) uPA-mediated active tumour targeting increased the intracellular drug concentration. (ii) CaP-mediated lysosomal membrane rupture resulted in lysosomal escape, along with (iii) the release of the BRCA1 siRNA to inhibit the DNA repair pathway. (iv) SiRNA and reduction of Pro-Pt to Pt synergistically induced irreversible DNA damage in TNBC cells. *siRNA* small interfering RNA, *TNBC* triple-negative breast cancer (Reproduced with permission from reference [161]. Copyright 2019, American Chemical Society)

More recently, several groups have not only utilized various uPAR-targeted nanoplatforms as drug delivery systems to enhance the antitumor effect but also used uPAR-targeted nanoparticles (NPs) as targeted therapeutic imaging probes. Dong et al. successfully loaded BRCA1 small interfering RNA (siRNA), which block DNA repair, and the DNA-damaging agent Pro-Pt into a shell-core pH-sensitive platform (uPA-SP@CaP NPs) to increase the sensitivity of triple-negative breast cancer (TNBC) to chemotherapy. The NPs achieved dual tumour targeting through the passive enhanced permeability and retention (EPR) effect and active uPA peptide [161] (Fig. 3). Yang et al. engineered uPAR-targeted magnetic iron oxide nanoparticle (IONP)-encapsulated Dox conjugated with the ATF of uPA that delivered higher Dox loads and exerted a stronger inhibitory effect on breast cancer cell growth than nontargeted NPs. Moreover, these NPs have been used as targeted therapeutic imaging probes for monitoring drug delivery using magnetic resonance imaging (MRI) [162]. Miller-Kleinhenz et al. prepared Wnt/LRP5/6- and uPAR-targeted ultrasmall magnetic IONPs carrying Dox (iWnt-ATF_24_-IONP-Dox) that showed a stronger inhibitory effect than non/single-targeted IONPs on a human breast cancer patient-derived xenograft model and markedly inhibited Wnt/β-catenin signalling and the cancer stem-like phenotype by decreasing the levels of the Wnt ligand, CD44 and uPAR [163]. Lee et al. engineered ATF-mediated IONPs carrying gemcitabine (Gem) (ATF-IONP-Gem) to target uPAR-expressing tumour and stromal cells and overcome the tumour–stromal, which not only provided contrast enhancement in MRI of tumours, but also significantly inhibited the growth of orthotopic pancreatic cancer [164]. Gao et al. prepared uPAR-targeted PEGylated theranostic NPs (ATF-PEG-IONPs), and detected threefold higher intratumour accumulation (*i.p.* injection) than *i.v.* delivery; the IONPs were detected with NIR-830 labelling using noninvasive optical and MRI in an orthotopic pancreatic cancer model. Moreover, these IONPs carrying Cis or Dox (ATF-PEG-IONP-Cis or ATF-PEG-IONP-Dox) markedly inhibited tumour angiogenesis and tumour growth and reduced the production of malignant ascites [165].

Ahmed et al. developed multifunctional double-receptor-targeting IONPs [luteinizing hormone-releasing hormone (LHRH) peptide- and AE105 peptide-targeted IONPs, LHRH-AE105-IONPs] that simultaneously targeted the LHRH receptor (LHRH-R) and uPAR and exhibited a significant MRI contrast in PCa cells. Importantly, the IONPs carrying PTX (LHRH-AE105-IONPs-PTX) showed two times higher cell cytotoxicity than IONPs targeting a single molecule [166]. Park et al. prepared AE147 peptide-conjugated liposomes encapsulating DTX (DTX/AE Lipo) to actively target uPAR-overexpressing metastatic tumours. In MDA-MB-231 cells, DTX/AE-Lipo (IC_50_ 4.61 µg/mL) achieved better anticancer activity than free DTX (IC_50_ 7.18 µg/mL) or DTX/Lipo (IC_50_ 8.59 µg/mL). Additionally, AE147-conjugated liposomes showed improved tumour-targeting ability [167]. Belfiore et al. prepared anti-mitotic *N*-alkylisatin (*N*-AI)-loaded liposomes modified with plasminogen activator inhibitor type 2 (PAI-2/SerpinB2) to target uPA/uPAR. The liposomes showed a higher uptake in MDA-MB-231 cells than in MCF-7 cells and higher accumulation at the tumour site than the nontargeted liposomes [168]. Wang et al. prepared synthetic self-assembled NPs modified with the U11 peptide-lipid amphiphile, which showed an essentially tenfold higher transfection efficiency than scrambled peptide-targeted NPs in uPAR-positive DU145 cells [105]. Hong et al. employed a U11 peptide-decorated, pH-sensitive NP system by coencapsulating the U11 peptide-conjugated, pH-sensitive Dox prodrug (U11-Dox) and curcumin (Cur) (U11-Dox/Cur NPs), and this formulation displayed a higher cellular uptake and tumour accumulation than nontargeting NPs and inhibited tumour growth by 85% in vivo [169].

Our research group also developed β-elemene-loaded liposomes modified with ATF_24_ peptide (ATF_24_-PEG-Lipo-β-E); these liposomes showed better targeting efficiency and higher cytotoxicity than nondecorated liposomes and exerted a synergistic effect on inhibiting the growth of KU-19-19 bladder cancer with Cis [170]. Devulapally et al. successfully developed a uPA peptide (VSNKYFSNIHWGC)-conjugated, antisense-miR-21 and antisense-miR-10b coloaded PLGA-*b*-PEG-NPs (called uPA-Anti-miR-21-Anti-miR-10b-NPs) that simultaneously antagonized miR-21-induced inhibition of apoptosis and miR-10b-induced metastasis to achieve TNBC therapy [171]. Therefore, uPAR-targeted theranostic NPs have tremendous potential for future imaging and targeted therapeutic applications because they are capable of enhancing the therapeutic effect of NP drugs on various types of cancers. The uPAR-targeted nanoplatforms carrying therapeutic agents are summarized in Table 2.

**Table 2:** The uPAR-targeted nanoplatforms carrying therapeutic agents

Nano platform	Target	Drug	Imaging	Effect	References, year
uPA-SP@CaP NPs	uPA peptide, amino acid sequence: VSNKYFSNIHWGC (uPAR)	BRCA1 siRNA, Pro-Pt	Fluorescence imaging (Dir)	Improve anticancer efficacy of the TNBC (pH-responsive sequential release ability, lysosomal escape property, dual tumour targeting, and irreversible DNA damage behavior)	[161], 2019
ATF-IO-Dox	ATF (uPAR)	Dox	MRI	A marked inhibition of tumour cell growth in 4T1 and MDA-MB-231 cells	[162], 2008
iWnt-ATF_24_-IONP-Dox	iWnt, amino acid sequence: NSNAIKNKKHHH (Wnt/LRP5/6), ATF_24_, amino acid sequence: CHHHCLNGGTCVSNKYFSNIHWCNCPKK (uPAR)	Dox	NIR-830 dye for optical imaging	Strong tumour growth inhibition in a human chemo-resistant cancer patient-derived xenograft model (inhibited Wnt/β-catenin signaling and cancer stem-like phenotype of tumour cells; marked reduction of Wnt ligand, CD44 and uPAR)	[163], 2018
ATF-IONP-Gem	ATF (uPAR)	Gem	MRI	Inhibit the growth of orthotopic human pancreatic cancer xenografts in nude mice (overcoming the tumour stromal barrier)	[164], 2013
ATF-PEG-IONP-Cis or ATF-PEG-IONP-Dox	ATF (uPAR)	Cis or Dox	NIR optical imaging and MRI	Inhibit the growth of pancreatic tumours (*i.p.*); decrease proliferating tumour cells and tumour vessels; reduce the amount of ascites production	[165], 2017
LHRH-AE105-IONPs-PTX	LHRH (LHRH-R), AE105 (uPAR)	PTX	MRI	10 times reduction in the concentration of PTX required to achieve similar cytotoxic effect produced by the free drug (LHRH-R- and uPAR-overexpressing PC-3 cells)	[166], 2017
DTX/AE Lipo	AE147 (uPAR)	DTX	Fluorescence imaging	DTX/AE-Lipo (IC_50_ 4.61 µg/mL) achieves better anticancer activity than free DTX (IC_50_ 7.18 µg/mL) or DTX/Lipo (IC_50_ 8.59 µg/mL)	[167], 2021
PAI-2 *N*-AI liposomes	PAI-2 (uPAR)	*N*-alkylisatin	NA	An increased accumulation at the primary tumour site in an orthotopic MDA-MB-231 BALB/c-Fox1nu/Ausb xenograft mouse model	[168], 2020
U11 peptide targeted NPs	U11 peptide (uPAR)	Plasmid DNA	Fluorescence imaging (Rhodamine)	Transfection of uPAR positive DU145 cells is essentially tenfold higher compared to transfection achieved by NPs having a scrambled peptide sequence on their surface	[105], 2009
U11-Dox/Cur NPs	U11 peptide (uPAR)	Dox/Cur	Fluorescence imaging (Coumarin 6)	Inhibit the tumour growth to a level of 85%	[169], 2019
ATF_24_-PEG-Lipo-β-E	ATF_24_ (uPAR)	β-E	Fluorescence imaging (Did)	Combined with Cis, exert a synergistic effect on cellular apoptosis and cell arrest at the G2/M phase (dependent on the caspase-dependent pathway and Cdc25C/Cdc2/cyclin B1 pathways)	[170], 2020
uPA-Anti-miR-21-Anti-miR-10b-NPs	uPA peptide (VSNKYFSNIHWGC)	Antisense-miR-21, antisense-miR-10b	Optical bioluminescence imaging (MDA-MB-231-Fluc-eGFP cells)	40% reduction in tumour growth compared to scrambled peptide conjugated NPs treated mice (0.15 mg/kg)	[171], 2015

*siRNA* small interfering RNA, *TNBC* triple-negative breast cancer, *NIR* near infrared, *MRI* magnetic resonance imaging, *Gem* gemcitabine, *Cis* Cisplatin, *Dox* doxorubicin, *DTX* docetaxel, *Cur* curcumin, *PTX* paclitaxel, *β-E* β-elemene, *Cdc25C* cell division cyclin 25C, *Cdc2* cell division cycle protein 2, *Dir* 1,1′-dioctadecyl-3,3,3′,3′-tetramethylindotricarbocyanine iodide, *Did* 1,1′-dioctadecyl-3,3,3′,3′-tetramethylindodicarbocyanine perchlorate, *NPs* nanoparticles

## uPAR-targeted PDT/PTT platforms

Among anticancer treatments, PDT and PTT are widely regarded as promising cancer therapeutic strategies due to their unique advantages such as minor trauma, improved selectivity, remarkable spatial/temporal resolution and reduced side effects [172]. PDT depends on photosensitizers (PSs) that produce reactive oxygen species (ROS) upon light activation, and subsequently induce cell apoptosis [173]. PTT is a type of phototherapy that converts absorbed light to local heat in tumours using various nanomaterials such as gold nanorods, carbon nanohorns and graphene oxide, and thus induces cell death [174]. Recently, a variety of uPAR-targeted PDT/PTT strategies have been developed to enhance the therapeutic effect on malignant tumours and reduce systemic side effects.

**Fig. 4:**
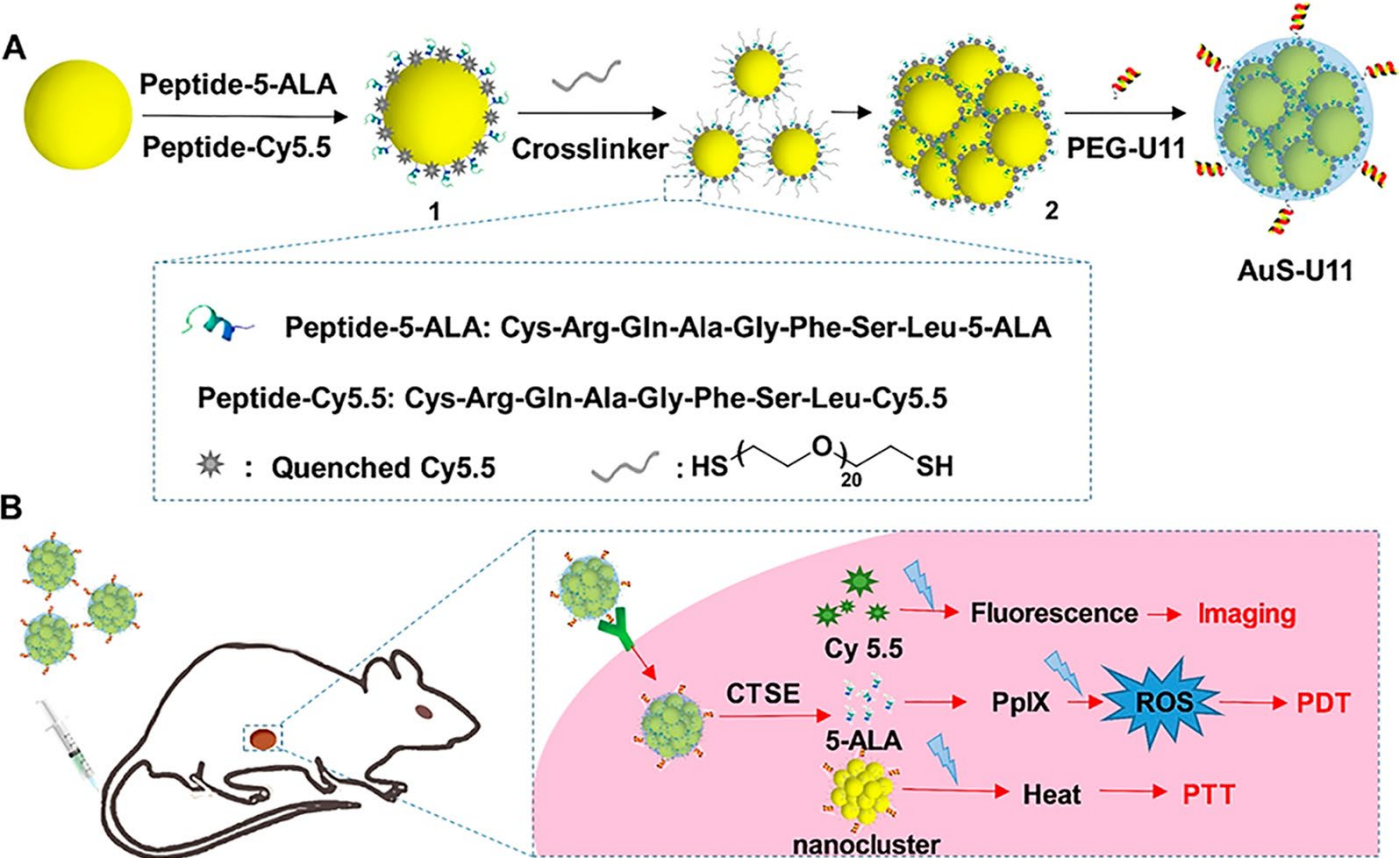
uPAR-targeted, CTSE-responsive gold nanoclusters as a PDT/PTT platform. **A** Schematic of the synthetic route used to produce the gold nanoclusters in three steps: CTSE-cleavable CRQAGFSL-5-ALA (Peptide-5-ALA, prodrug) and CRQAGFSL-Cy5.5 (Peptide-cy5.5) were covalently conjugated to the nanospheres, cross-linked with 1,9-nonanedithiol to produce spherical gold nanoclusters, and finally coated with the U11 peptide modified PEG layer to yield the uPAR-targeted, CTSE-responsive PDT/PTT platform. **B** Overview of image-guided PDT/PTT in therapeutic PDAC. Upon injection, the nanoclusters first targeted the pancreatic tumour tissue. The selective cleavage of the CTSE-sensitive peptide activated the fluorescence signal of the NIR cyanine dye Cy5.5 to guide PDT/PTT therapy using confocal laser endomicroscopy. *CTSE* cathepsin E, *5-ALA* 5-aminolevulinic acid, *Cy5.5* cyanine 5.5, *PDAC* pancreatic ductal adenocarcinoma, *NIR* near infrared, *PEG* polyethylene glycol, *ROS* reactive oxygen species (Reproduced with permission from reference [172]. Copyright 2017, Pergamon)

Li et al. engineered a U11 peptide modified gold nanocluster platform carrying the cathepsin E (CTSE)-sensitive PDT prodrug/imaging agent CRQAGFSL-5-aminolevulinic acid (5-ALA)/-cyanine 5.5 (Cy5.5) (AuS-U11), which showed excellent efficacy with endomicroscopy-guided PTT/PDT through the combination of active tumour targeting and enzyme-triggered release of 5-ALA and Cy5.5 in a PANC1-CSTE orthotopic tumour model [172] (Fig. 4). Li et al. prepared a human ATF-decorated human serum albumin (HSA) carrying the photosensitizer monosubstituted β-carboxy phthalocyanine zinc (CPZ) (hATF-HSA:CPZ), and detected a greater tumour accumulation than HSA:CPZ using fluorescent molecular tomography (FMT) by targeting uPAR on the tumour cell surface to subsequently achieve highly efficient photodynamic killing of tumours in an H22 tumour model [175]. Zhou et al. also generated a CPZ loaded mouse ATF-HSA (mATF-HSA:CPZ) that achieved an enhanced murine tumour targeting ability and an enhanced PDT efficacy compared with hATF-HSA:CPZ [176]. Based on this information, the author further developed CPZ-loaded uPAR-targeted receptor-responsive NPs (ATF-HSA:CPZ@RRNP) with a diameter of ~ 40 nm. Interestingly, ATF-HSA:CPZ@RRNP, but not the nontargeting NPs, disintegrated into 7.5 nm fragments and released its cargo in the presence of uPAR. These NPs also exhibited higher cytotoxicity toward H1299 cells and greater tumour accumulation and antitumor effects on the H22 tumour model than HSA:CPZ@RRNP [177]. Chen et al. designed an active targeting phototherapeutic agent by conjugating zinc phthalocyanine (ZnPc) with ATF (ATF-ZnPc), which not only exhibited a high binding affinity and potent PDT activities to uPAR-positive U937 and H1299 cells, but also was used as a biomarker for the noninvasive imaging of tumours [178].

In addition, Yu et al. developed uPAR-targeted polyetherimide-AE105 peptide (P-AE105) conjugated gold nanostars (GNS) carrying an iridium (Ir) complex that exerted enhanced anti-TNBC effects through the ROS-induced p53 apoptotic pathway, and showed excellent PT/photoacoustic (PA)/X-ray computed tomography (CT) imaging properties [179]. Hu et al. constructed an AE105 peptide conjugated gold nanorod mesoporous silica heterostructure loaded with Cis and Avastin (Cis-AuNRs@SiO_2_-Avastin@PEI/AE105), and observed a prominent photodynamic killing effect and anti-angiogenic activity by targeting uPAR and smart light-controlled drug release in a HeLa tumour model [180]. Zuo et al. designed and constructed AE105-decorated dendritic mesoporous silica NPs (DMSN) encapsulating photonic active ultrasmall Cu_2−x_S NPs and the sonosensitizer Rose Bengal (RB) (Cu_2−x_S-RB@DMSN-AE105, abbreviated as CRDA) for OSCC-targeting and synergetic PTT/sonodynamic therapy (SDT) [181]. Hu et al. also developed anti-uPAR antibody and indocyanine green (ICG)-modifed gold nanoshells (uIGNs), and achieved a 25% higher median survival rate and complete tumour ablation than clinical iodine-125 (^125^I) interstitial brachy-therapy (IBT-125-I). Furthermore, uIGNs prevented pancreatic tumour metastasis, as evidenced by real-time monitoring of metastatic tumours (less than 2 mm) using CT and NIR imaging [182]. The uPAR-targeted PDT/PTT platforms are summarized in Table 3.

**Table 3:** The uPAR-targeted PDT/PTT platforms

uPAR-targeted PDT/PTT platform	Target	Photosensitizer and drug	Imaging	Effect	References, year
AuS-U11 for confocal laser endomicroscopy-guided PTT/ PDT	U11 peptide (uPAR)	PTT-carrier gold nanocluster, CRQAGFSL-5-ALA, CRQAGFSL-Cy5.5	Fluorescence images (enzyme-triggered release of NIR fluorescent dye Cy5.5)	Better synergistic therapeutic effects as well as the reduced side effects in normal pancreas tissue (human pancreatic tumour cell line PANC1-CSTE and its orthotopic tumour model)	[172], 2017
hATF-HSA:CPZ	hATF (uPAR)	CPZ	FMT imaging (CPZ, 0.08 μmol/kg or 0.05 mg/kg)	A significant reduced tumour growth rate (H22 tumour-bearing Kunming mice model)	[175], 2014
mATF-HSA:CPZ	mATF (uPAR)	CPZ	FMT imaging (CPZ, 0.05 mg/kg)	A higher tumour killing efficacy than hATF-HSA:CPZ (H22 tumour-bearing mouse model)	[176], 2015
ATF-HSA: CPZ@RRNP	ATF (uPAR)	CPZ-loaded receptor-responsive nanoparticles	FMT imaging (CPZ, 0.05 mg/kg)	Higher uptake and cytotoxicity (H1299 lung cancer cells), higher tumour accumulation and better antitumour effect (H22 tumour-bearing mice), lower CPZ concentration (liver, kidney, spleen, lung, and heart)	[177], 2019
ATF-ZnPc	ATF (uPAR)	ZnPc	FMT imaging (ATF-ZnPc, 0.4 μmol/kg)	Potent PDT activities and enhanced antitumour activity (U937 and H1299 cells and H22 tumour-bearing mice)	[178], 2014
GNS@Ir@P-AE105	AE105 (uPAR)	GNS, Ir complex	PT/PA/X-ray CT trimodal imaging	Combinational photothermal-chemotherapeutic efficiency against TNBC via a ROS-induced p53 apoptotic pathway	[179], 2020
Cisplatin-AuNRs@SiO_2_-Avastin@PEI/AE105	AE105 (uPAR)	Gold nanorod mesoporous silica heterostructure, cisplatin, Avastin	Photothermal imaging (3 mg/kg)	Photodynamic activity via induction of ROS overproduction-mediated cell apoptosis, suppresses HeLa tumour growth and angiogenesis	[180], 2019
Cu_2−x_S-RB@DMSN-AE105	AE105 (uPAR)	Cu_2−x_S NPs, Rose Bengal	Infrared thermal imaging	Synergetic PTT/SDT nanotherapeutics against the OSCC both in vitro and in vivo, a prominent tumour inhibition rate of 103.4%	[181], 2020
uIGNs	Anti-uPAR antibody	ICG modifed gold nanoshells	CT and optical imaging (bioluminescence imaging and fluorescence imaging)	25% higher median survival rate of IPTT and complete tumour ablation by one-time intervention, inhibit pancreatic tumour metastasis	[182], 2017

*PDT* photodynamic therapy, *PTT* photothermal therapy, *5-ALA* 5-aminolevulinic acid, *Cy5.5* cyanine 5.5, *HSA* human serum albumin, *CPZ* mono-substituted β-carboxy phthalocyanine zinc, *FMT* fluorescent molecular tomography, *ZnPc* zinc phthalocyanine, *SDT* sonodynamic therapy, *OSCC* oral squamous cell carcinoma, *CT* computed tomography, *PT* photothermal, *PA* photoacoustic, *GNS* gold nanostars, *Ir* iridium, *ICG* indocyanine green, *ROS* reactive oxygen species, *IPTT* interventional PTT

## uPAR-targeted oncolytic virotherapy

Oncolytic virotherapy is an emerging platform that represents a novel frontier for cancer treatment. Redirecting viral tropism to specific tumour targets is a promising strategy in the field of oncolytic viruses, which may increase safety and inhibit distant metastases of tumours [183]. Recently, some retargeted oncolytic measles viruses (MVs) against uPAR have been developed.

MV-h-uPA or MV-m-uPA, an Edmonston vaccine strain of oncolytic MVs constructed by the ATF of human or murine uPA and mutant MV-H glycoprotein, was able to replicate, and induce cytotoxicity in a species-specific manner. In vivo, MV-h-uPA successfully inhibited tumour growth (inhibition rate of 76% at Day 39), prolonged survival (70% survival rate at Day 80) and reduced metastatic progression in an MDA-MB-231 tumour model [184]. In addition, MV-m-uPA increased the death of murine mammary (4T1) and colon (MC-38 and CT-26) tumour cells overexpressing uPAR. MV-m-uPA also significantly enhanced the anticancer effects and prolonged survival in CT-26 and 4T1 tumour models [185], and delayed 4T1 lung metastasis progression. In conclusion, MV-uPA is a novel oncolytic MV associated with potent and specific antitumour and antimetastatic effects [186].

Tumour stroma-selective targeting by uPAR retargeted MVs is also associated with enhanced antitumour effects. For example, MV-m-uPA inhibits breast cancer cell proliferation by selectively targeting fibroblasts, and delays tumour progression and prolongs survival in mice bearing a human MDA-MB-231 tumour model [187]. MV-CD46-muPA, a dual-targeted oncolytic MV that simultaneously targets murine stromal (via uPAR) and human cancer cells (via CD46), markedly enhances antitumour effects on the HT-29 tumour model compared to CD46-targeted MV alone. The improved effect was associated with the modulation of viral deposition, cell cycle and metabolic pathways, increased apoptosis and decreased murine stromal [188].

## uPAR-targeted gene therapy technologies

The development of efficient and reliable methods to generate precise, targeted changes in the genome of living cells is a long-standing goal for biomedical researchers. In uPAR-targeted gene therapy technologies, adenovirus-mediated antisense uPAR therapy first emerged as an effective tool for cancer treatment. For example, an adenoviral vector containing the uPAR antisense sequence (Ad-uPAR), an adenovirus containing uPAR antisense and p16 sense expression cassettes (Ad-uPAR/p16), an adenovirus expressing antisense uPAR and uPA sequences (Ad-uPAR-uPA), an adenovirus vector containing antisense uPAR and cathepsin B sequences (Ad-uPAR-Cath B), and an adenovirus expressing antisense uPAR and MMP-9 sequences (Ad-uPAR-MMP-9) were all successfully constructed and inhibited tumour growth and metastasis in gliomas and lung cancer models [189–193].

Subsequently, RNA interference (RNAi) technologies, including siRNAs and short hairpin RNAs (shRNAs) targeting uPAR (siRNAs against uPAR, siRNAs against uPAR and cathepsin B, siRNAs against uPA and uPAR, shRNAs against uPAR, and shRNAs against uPA and uPAR), were developed to prevent tumour progression. Compared with siRNAs/shRNAs targeting uPAR, siRNAs targeting uPAR and uPA or siRNAs targeting uPAR and cathepsin B exerted a better antitumor effect by inhibiting tumour cell proliferation, migration and invasion and angiogenesis and promoting tumour cell apoptosis [70, 194–198].

Recently, a new tool based on bacterial Cas9 from *Streptococcus pyogenes* has generated a considerable level of excitement. The RNA-guided CRISPR/Cas9 system is a powerful RNA-guided genome editing tool that utilizes a guide RNA (gRNA) to cleave the desired sequence in the genome and remove existing genes or add new genes. Due to the advantages of being fast, precise, and highly efficient, targeting uPAR with CRISPR/Cas9 technology has been successfully applied in a variety of malignant tumours to enhance the treatment effect [98]. Targeting uPAR in Neuro 2A cells using CRISPR/Cas9 decreases cell proliferation (~ 60%) and the number of Ki-67-positive cells by activating caspase-3, cleaving poly(ADP-ribose) polymerase-1 (PARP-1), and inhibiting tropomyosin receptor kinase C (TrkC) sactivity and AKT phosphorylation [199]. Wang et al. also targeted uPAR using CRISPR/Cas9 technology to suppress the proliferation, migration and invasion of HCT8/T and KB_V200_ cells. Furthermore, uPAR knockout inhibited MDR to 5-FU, Cis, DTX, and Dox [98]. Biagioni et al. also knocked out uPAR using the CRISPR/Cas9 system in human melanoma A375p and A375M6 cells and colon cancer HCT116 cells, inducing extensive glycolytic and oxidative phosphorylation reprogramming by blocking the glycolytic pathway while enhancing the mitochondrial spare respiratory capacity [200]. They also reported that uPAR deficiency mediated by CRISPR/Cas9 induced a stem-like phenotype, but uPAR knockout completely eliminated tumorigenesis [201].

## uPAR-targeted monoclonal antibody therapy

A variety of monoclonal antibodies targeting uPAR have been developed, and exert antitumor effects by blocking the uPA/uPAR interaction or inhibiting the interactions between uPAR and integrin, EGFR, FPR, and Vn. The 2G10 antibody binds tightly to uPAR (Fab *K*_*d*_ = 10 × 10^–9^; IgG *K*_*d*_ = 2 × 10^–12^) by forming a stable complex with uPAR and disrupting the uPA/uPAR interaction. LeBeau et al. found that 30 mg/kg 2G10 IgG prevents the growth of TNBC, and ^177^Lu-labelled 2G10 completely eliminates tumours in orthotopic breast cancer models [202]. Harel et al. further prepared the antibody–drug conjugate 2G10-RED-244-MMAE to treat TNBC, and the tumour volume was significantly reduced [203]. Duriseti et al. identified a series of monoclonal antibodies that bind uPAR, including 2G10, 2E9 and 3C6. The 2G10 and 2E9 antibodies inhibited the uPA/uPAR interaction, whereas 3C6 inhibited the uPAR/β1 integrin interaction. Additionally, 3C6 abrogated uPAR/β1 integrin-mediated adhesion to Vn and fibronectin and exerted a synergistic effect with 2G10 on inhibiting invasion in H1299 cells [204].

ATN-658 is a humanized monoclonal antibody that binds to the D2D3 region of uPAR with high affinity (*K*_*d*_ ≈ 1 nmol/L), and the binding of ATN-658 to uPAR is not affected by the binding of uPA to uPAR. ATN-658 mainly inhibits the activation of downstream signalling pathways by inhibiting the uPAR/integrin interaction. ATN-658 inhibits the growth and liver metastasis of pancreatic cancer in situ and completely inhibits retroperitoneal infiltration; the antitumour effect is more obvious when this antibody is combined with Gem [65]. ATN-658 also significantly inhibits the growth of human colorectal cancer in the liver, and prevents the growth, migration, invasion and bone metastasis of prostate cancer [205, 206]. In addition, ATN-658 inhibits the metastasis of ovarian cancer and reduces the uPAR/α5-integrin interaction, and the tumour suppression rate is higher when it is combined with PTX [207]. ATN-658 significantly reduces the growth of MDA-MB-231 breast tumours, and when combined with Zometa, it significantly reduces the number of bone lesions caused by breast cancer by inhibiting the activity of osteoclasts [208]. Li et al. also prepared the monoclonal antibody ATN-615 that binds uPAR with high affinity (*K*_*d*_ ≈ 1 nmol/L) and does not block the uPA/uPAR interaction [209]. ATN-292, isotype IgG1κ, decreases the migration of human pancreatic carcinoma L3.6pl cells (70% ± 8%) by inhibiting the binding of uPA to uPAR [65].

Two antibodies, mAb R3 and mAb R5, are competitive and noncompetitive inhibiters of the uPA/uPAR interaction, respectively. mAb R5 binds the preformed complex and promotes the dissociation of the uPA/PAR complex, while mAb R3 does not promote the dissociation of the preformed complex [210]. Pass et al. developed an anti-muPAR murine mAb (mR1) that interferes with the muPA/muPAR interaction on P388D.1 cells with an IC_50_ of 0.67 nM [211]. A monoclonal antibody against human uPAR, mAb 3936, also inhibits hepatocyte growth factor (HGF)-mediated HepG2 and Hep3B cell invasion in a dose-dependent manner [212]. The mAb 8B12, a specific inhibitor that blocks the uPAR/Vn interaction, significantly decreases tumour growth by increasing cell apoptosis and reducing cell proliferation in a prostate cancer model. A crystal structure of the uPAR-8B12 complex showed that the structural epitope for 8B12 is located at the D2–D3 domain interface on the surface of uPAR [213].

## uPAR-targeted tumour immunotherapy

As an innovative treatment method, tumour immunotherapy has shown potential to fight cancer by modulating the immune system, such as checkpoint inhibitors and adoptive cellular therapy using CAR T-cell [214]. Based on the high expression of uPAR on the surface of tumour cells, some researchers have explored the combination of CAR T-cell immunotherapy and uPAR targeting to treat uPAR-expressing malignancies or the use of uPAR as a target to induce immune-mediated clearance of uPAR-positive tumour cells by constructing ARMs.

## uPAR-targeted CAR T-cell immunotherapy

CARs are synthetic receptors that contain an extracellular single-chain variable fragment (scFv), a hinge region that provides flexibility to the scFv, a transmembrane domain, and intracellular signalling/activation domain(s) [215, 216]. CAR T-cell immunotherapy, extracts the patient’s own key immune T-cells and embeds them with a CAR, that recognizes tumour cell surface antigens while activating T-cells to kill tumour cells. CAR T-cell immunotherapy has achieved remarkable success in treating refractory B-cell malignancies [217]. In recent years, some researchers have combined ATF and CAR T-cells to treat solid tumours with high uPAR expression. Wang et al. designed anti-uPAR CAR (ATF-CAR) T-cells constructed by combining an antigen recognition domain with ATF to transduce T-cells, and this treatment exhibited strong cytotoxicity toward uPAR-expressing ovarian cancer cells and released higher levels of Th1 cytokines [interferon-γ (IFN-γ), tumour necrosis factor (TNF) and interleukin-2 (IL-2)] and granzyme B than control T-cells [218]. Pathologically, cellular senescence may lead to a variety of diseases including cancer. Given the contribution of senescence to tumorigenesis, Amor et al. also developed an anti-uPAR CAR T-cells (m.uPAR-h.28z CAR T cells) by linking an anti-murine uPAR single chain variable fragment and human CD28 costimulatory and CD3ζ signalling domains to transduce human T-cells that efficiently cleared uPAR-expressing KP lung cancer cells, accompanied by increased secretion of granzyme B and IFN-γ. They also markedly prolonged survival and induced a significant decrease in the number of senescent tumour cells, accompanied by increased infiltration of CD4^+^ and CD8^+^ T cells in a mouse model of orthotopic KP lung adenocarcinoma [219].

## uPAR-targeted ARMs

ARMs are antibody-binding molecules that exert antitumour effects by delivering endogenous antibodies to tumour tissues and destroying tumour cells via the activated immune system [220]. Jakobsche et al. designed and synthesized an antibody-recruiting complex ARM-U1 by attaching chloromethyl ketone 2 and 2,4-dinitrophenyl (DNP) to the active site of uPA that mediated both antibody-dependent cellular phagocytosis (ADCP) and antibody-dependent cellular cytotoxicity (ADCC) against uPAR-expressing cancer cells [221]. The authors further designed a second-generation ARM-U2 by replacing the uPA protein with a molecule of IPR-803. ARM-U2 also induced both ADCP and ADCC, and achieved a tumour growth inhibition of approximately 90% compared to PBS treatment in the B16-uPAR mouse allograft model. They also reported a cocrystal structure of the ARM-U2/uPAR complex for the first time. In conclusion, uPAR-specific CAR T cells and ARMs are promising immunotherapies that not only block the uPA/uPAR interaction, but also achieve immune-mediated cell death by targeting uPAR-expressing tumour cells [222]. In addition, Hu et al. developed an antibody-like molecule, ATF-Fc, formed by linking ATF and the human IgG1 Fc fragment. ATF-Fc inhibits the growth and metastasis of MCF-7 breast cancer and BGC-823 gastric cancer cells by destroying the interaction of uPA/uPAR and inhibiting tumour angiogenesis [223]. Zhou et al. further showed that the combination of ATF-Fc and trastuzumab better inhibits the growth and metastasis of HER-2-positive breast cancer cells by interfering with the uPA/uPAR and HER-2 pathways [224].

## Concluding remarks

uPAR is an attractive target for the treatment of cancer because it appears to be expressed at high levels in tumours but low levels in normal tissue. uPAR also plays a comprehensive role in the development of tumours and is closely related to tumour proliferation and apoptosis, invasion and metastasis, prognosis, and tumour MDR, providing a basis for the development of multiple therapeutics agents targeting this protein. This review has summarized multiple new applications of uPAR as a target in nanoplatforms carrying therapeutic agents, PTT/PDT platforms, oncolytic virotherapy, gene therapy technologies, monoclonal antibody therapy and tumour immunotherapy in recent years. The development of therapeutic strategies that target tumours via uPAR recognition has proven its potential in animal models, but no uPAR-targeted therapeutic agents have been developed or evaluated in cancer clinical trials to date. Recently, ATN-658 has been humanized (huATN-658) and is awaiting clinical translation; and phase I clinical trials with ^64^Cu-DOTA-AE105 are being conducted to diagnose aggressive cancers and determine cancer aggressiveness. These two agents are expected to be administered to patients in the future.

Among uPAR-targeted therapeutic strategies, uPAR-targeted nanoplatforms also have great potential to achieve translation from laboratory findings to the clinic. Based on the high expression of uPAR on the surface of a variety of tumour cells, uPA/ATF/AE105/AE147/PAI-2/U11 modified nanoplatforms provide the possibility of reducing or overcoming the therapeutic limitations of conventional chemotherapy or PTT/PDT through targeted delivery to tumour cells without obvious toxicity to healthy tissue. Moreover, recent studies have a key role for the tumour microenvironment in promoting tumour proliferation, invasion and metastasis [225]. uPAR expression is not confined to tumour cells and is found on tumour-associated cell types, including macrophages, endothelial cells and fibroblasts. The development of uPAR-targeted stroma-breaking or stroma-penetrating NPs may allow therapeutic agents to overcome stromal barriers and reach tumour cells, which is highly likely to improve the therapeutic effect of current treatment agents and may provide better therapeutic options for patients to reduce tumour-associated metastasis.

## Data Availability

Not applicable.

## Abbreviations

uPAR: Urokinase-type plasminogen activator receptor
ECM: Extracellular matrix
MDR: Multidrug resistance
PDT: Photodynamic therapy
PTT: Photothermal therapy
uPA: Urokinase plasminogen activator
MMP: Matrix metalloproteinase
Vn: Vitronectin
EGFR: Epidermal growth factor receptor
GPCRs: G-protein coupled receptors
FAK: Focal adhesion kinase
MAPK: Mitogen-activated protein kinase
AKT: Protein kinase B
FPR: Formyl peptide receptor
HER-2: Human epidermal growth factor receptor-2
PAI: Plasminogen activator inhibitor
ERK: Extracellular regulatory protein kinase
Cis: Cisplatin
DTX: Docetaxel
Dox: Doxorubicin
PEG: Polyethylene glycol
DT: Diphtheria toxin
TNBC: Triple-negative breast cancer
NPs: Nanoparticles
IONP: Iron oxide nanoparticle
MRI: Magnetic resonance imaging
*N*-AI: *N*-Alkylisatin
HSA: Human serum albumin
CPZ: Mono-substituted β-carboxy phthalocyanine zinc
MVs: Oncolytic measles viruses
siRNA: Small interfering RNA
shRNA: Short hairpin RNA
CRISPR: Clustered regularly interspaced short palindromic
Cas9: CRISPR-associated protein-9 nuclease
CAR: Chimeric antigen receptor
ARMs: Antibody-recruiting molecules
